# tRNAs as regulators of biological processes

**DOI:** 10.3389/fgene.2014.00171

**Published:** 2014-06-11

**Authors:** Medha Raina, Michael Ibba

**Affiliations:** ^1^Department of Microbiology, The Ohio State Biochemistry Program, The Ohio State UniversityColumbus, OH, USA; ^2^Center for RNA Biology, The Ohio State UniversityColumbus, OH, USA

**Keywords:** amino acid, protein synthesis, regulation, transfer RNA, translation

## Abstract

Transfer RNAs (tRNA) are best known for their role as adaptors during translation of the genetic code. Beyond their canonical role during protein biosynthesis, tRNAs also perform additional functions in both prokaryotes and eukaryotes for example in regulating gene expression. Aminoacylated tRNAs have also been implicated as substrates for non-ribosomal peptide bond formation, post-translational protein labeling, modification of phospholipids in the cell membrane, and antibiotic biosyntheses. Most recently tRNA fragments, or tRFs, have also been recognized to play regulatory roles. Here, we examine in more detail some of the new functions emerging for tRNA in a variety of cellular processes outside of protein synthesis.

## INTRODUCTION

tRNAs are important players in the protein synthesis pathway, linking the genetic code with the amino acid sequence of proteins. tRNAs are composed of 73–90 nucleotides and have a characteristic cloverleaf secondary structure made up of the D-loop, T loop, variable loop, and the anticodon loop. The tRNA further folds into an L-shaped tertiary structure through coaxial stacking of the T and D loops. To function as a substrate in protein synthesis, tRNA is charged with an amino acid by its cognate aminoacyl-tRNA synthetase. The aminoacyl-tRNA (aa-tRNA) thus formed serves as a substrate and participates in the chemistry of peptide bond formation in the process of protein synthesis. Beside this well-known canonical role during protein biosynthesis, tRNAs have been shown to perform additional functions such as acting as signaling molecules in the regulation of numerous metabolic and cellular processes in both prokaryotes and eukaryotes. Aminoacylated tRNAs have also been implicated as substrates for non ribosomal peptide bond formation in the case of cell wall formation, protein labeling for degradation, modification of phospholipids in the cell membrane, and antibiotic biosynthesis. Due to their universally conserved L-shaped three-dimensional conformation, which is stabilized by extensive secondary and tertiary structural contacts and modifications, tRNA molecules are among the most stable RNAs in a cell and are considerably more robust than mRNAs (Gebetsberger and Polacek, 2013). For a long time, tRNA fragments were considered as non-functional degradation intermediates, but have now been recognized to be major RNA species in human cells for which regulatory roles are beginning to be discovered. It was also recently shown that tRNAs can act as an effective scavenger of cytochrome *c*, consistent with a role in regulating apoptosis. With new functions still emerging for tRNA, in this review we examine some of the many “non-protein synthesis” roles of tRNA in the cell.

## ROLES OF tRNA IN GENE EXPRESSION

While aminoacyl-tRNAs have been implicated in many roles outside translation, several important functions of tRNA have been found not to require the aminoacyl form (aa-tRNA). Uncharged tRNAs have been shown to regulate global gene expression in response to changes in amino acid pools in the cell. Bacteria have adopted various strategies to adapt to external stresses, of which the most-studied global regulatory system is the stringent response. Stringent response is mediated through the production of the alarmone 5′-diphosphate 3′-diphosphate guanosine (ppGpp) and 5′-triphosphate 3′-diphosphate guanosine (pppGpp) which were first discovered by Cashel and Gallant (1969) in *Escherichia coli* as a response to amino acid starvation. *E. coli* uses two pathways for the synthesis of ppGpp dependent on RelA and SpoT. RelA is a ribosome-associated (p)ppGpp synthase which senses the presence of uncharged tRNAs that accumulate at the ribosome A site as a result of amino acid limitation. The presence of the uncharged tRNA acts as an effector molecule, stalling protein synthesis and activating RelA which then synthesizes pppGpp and ppGpp by phosphorylation of GTP or GDP using ATP as the phosphate donor (Haseltine and Block, 1973; Sy and Lipmann, 1973). ppGpp was recently shown to bind at an interface of ω and β′ subunits of RNA polymerase, thereby acting as an allosteric effector to inhibit global gene transcription, while stimulating the expression of only a few genes related to the synthesis of amino acids (Ross et al., 2013). rRNA and tRNA synthesis are primarily inhibited, resulting in the global downregulation of bacterial metabolism. SpoT is a bifunctional (p)ppGpp synthase and hydrolase, which presumably regulates the (p)ppGpp level in response to nutrient deficiency. The mechanism by which SpoT senses starvation and synthesizes ppGpp is unclear (Magnusson et al., 2005). Many other bacterial species including *Bacillus subtilis* contain only one RelA-SpoT homolog, designated as Rel, which possesses both (p)ppGpp synthase and hydrolase activities. RelA-SpoT homologs have also been detected in plants (Givens et al., 2004). Two *Bacillus subtilis* genes, yjbM and ywaC, were found to encode a novel (p)ppGpp synthase that corresponds to the synthase domain of RelA-SpoT family members while having a different mode of action (Nanamiya et al., 2008).

Another mechanism by which bacteria regulate gene expression using uncharged tRNA as the effector molecule has been demonstrated in *B. subtilis* and other Gram-positive bacteria. In these organisms, the expression of aminoacyl-tRNA synthetase genes and genes involved in amino acid biosynthesis and uptake is regulated by the T box control system (reviewed in Green et al., 2010). Regulation by the T box mechanism most commonly occurs at the level of transcription attenuation (Henkin and Yanofsky, 2002). The 5′ untranslated regions of regulated genes contain a 200–300 nt conserved sequence and structural element (a G + C-rich helix followed by a run of U residues) that serves as an intrinsic transcriptional terminator and can also participate in formation of an alternate, less stable antiterminator structure. During amino acid starvation, binding of a specific uncharged tRNA stabilizes the antiterminator and in doing so prevents formation of the terminator helix. The T box binds specific uncharged tRNA at two conserved sites: the anticodon of the tRNA interacts with the codon sequence of the specifier loop (SL) in the 5′-UTR, while the 3′ acceptor end interacts with the UGGN sequence found in the antiterminator bulge, thus stabilizing the structure of the antiterminator and preventing the formation of the competing terminator. RNA polymerase then continues past the terminator region and transcribes the full-length mRNA. The N residue in the antiterminator bulge varies with the corresponding position of the tRNA. Both charged and uncharged tRNAs can interact with specifier sequence in the 5′-UTR; however the presence of the amino acid at the 3′ end of a charged tRNA prevents the interaction of its 3′ end with the antiterminator bulge region; and allows formation of the terminator hairpin that results in premature termination of transcription (Grundy et al., 2005). Recently a unique mechanism of tRNA-dependent regulation at the transcriptional level was discovered. Saad et al. (2013) found a two-codon T-box riboswitch binding two tRNAs in *Clostridium acetobutylicum*. This T-box regulates the operon for the essential tRNA-dependent transamidation pathway and harbors an SL with two potential overlapping codon positions for tRNA^Asn^ and tRNA^Glu^. Both tRNAs can efficiently bind the SL *in vitro* and *in vivo*. This feature allows the riboswitch to sense two tRNAs and balance the biosynthesis of two amino acids (Saad et al., 2013). Regulation at the level of translation initiation has also been demonstrated for T box riboswitches in certain bacteria (Fuchs et al., 2006). Translationally regulated leader RNAs include an RNA element with the ability to sequester the Shine-Dalgarno (SD) sequence by pairing with a complementary anti-SD (ASD) sequence. Binding of uncharged tRNA stabilizes a structure analogous to the antiterminator that includes the ASD sequence, and formation of this alternate structure releases the SD sequence for binding of the 30S ribosomal subunit, thereby enabling translation of mRNA coding for proteins involved in amino acid biosynthesis (Green et al., 2010).

Uncharged tRNAs also function as regulators in eukaryotes. In amino-acid-starved yeast and mammalian cells, uncharged tRNA activates a protein kinase named Gcn2p whose regulatory sequences include the amino terminal region, a pseudo kinase domain, protein kinase region, histidyl-tRNA synthetase (HisRS)-related region and the c-terminal dimerization and ribosome binding sequences. The tRNA has been shown to bind to the HisRS like regulatory domain, thereby activating Gcn2p which in turn phosphorylates eIF2, a protein involved in binding GTP and Met-tRNA_i_^Met^ and forming the ternary complex required for translation initiation (Wek et al., 1995). The activated Gcn2p phosphorylates the α subunit of eIF2 at serine 51, lowering its activity and thereby reduces global protein synthesis. Gcn2p was shown to bind several types of uncharged tRNA with similar affinities but showed a reduced affinity for the charged form of a tRNA, implying that Gcn2 can discriminate between charged and uncharged forms of tRNA. (Dong et al., 2000). It was recently proposed that in the inactive form of Gcn2 present in non-starvation conditions, association with the substrate eIF2 is prevented by binding of the HisRS-like domain and C-term to the PK domain of Gcn2 thereby sequestering the substrate binding cleft. However, under starvation conditions, uncharged tRNA binds to Gcn2, at both the HisRS and C-term domains thereby producing conformational changes which open up the substrate binding cleft in the PK domain by releasing the HisRS-like domain and the C-terminal portion of Gcn2p, from inhibitory interactions with the PK domain, which allows eIF2 binding and phosphorylation (Qiu et al., 2001).

It has been proposed that discrimination between the charged and uncharged tRNA by Gcn2p occurs via an analogous mechanism of RelA protein activation as observed in *E. coli* by the presence of uncharged tRNA at the decoding (A) site on translating ribosomes. The activation of Gcn2p by uncharged tRNA requires its association with the ribosome via its C-terminal region and also, interactions between the N terminus of Gcn2p and the Gcn1p–Gcn20p protein complex which is also associated with the ribosome. Gcn1p, has been proposed to facilitate the eviction of uncharged tRNA from the A site and its transfer from the A site to the HisRS-like domain in Gcn2p for kinase activation and the Gcn1p-Gcn20p complex has also been implicated to increase the binding of uncharged tRNA to ribosomes. The importance of the Gcn1p–Gcn20p complex in Gcn2p activation was shown by the Hinnebusch group, who demonstrated that deletion of GCN1 blocks eIF2 phosphorylation by Gcn2p (Marton et al., 1993). The activation of eIF2 by an uncharged tRNA at the A site of the ribosome could explain how starvation of a single amino acid can activate Gcn2p, even though it cannot discriminate between uncharged tRNA species in cells starved for only one amino acid (Marton et al., 1997; Garcia-Barrio et al., 2000; Sattlegger and Hinnebusch, 2000). In yeast, phosphorylation of eIF2, allows for selected mRNAs such as *GCN4* to be translated. Elevated levels of Gcn4, which acts as a transcription factor, stimulate the expression of genes involved in amino acid biosynthesis (reviewed in Hinnebusch, 2005). In comparison to *S. cerevisiae*, which has a single eIF2α kinase, Gcn2p, mammalian cells have expanded this stress response pathway to include additional eIF2α kinases, which each respond to different environmental stresses. Analogous to yeast, phosphorylation of mammalian eIF2α leads to a block in global translation, accompanied by induced translational expression of ATF4 and ATF5, transcription factors related to Gcn4p (Harding et al., 2000; Lu et al., 2004; Vattem and Wek, 2004; Zhou et al., 2008).

The above mechanisms demonstrate that under certain nutritional stresses, the aminoacylation levels of tRNAs change and the accumulated uncharged tRNAs participate in numerous biological pathways that regulate global gene expression levels, helping the organism to survive under adverse conditions.

## AMINOACYL-tRNAs AS NON-RIBOSOMAL SUBSTRATES

**FIGURE 1:**
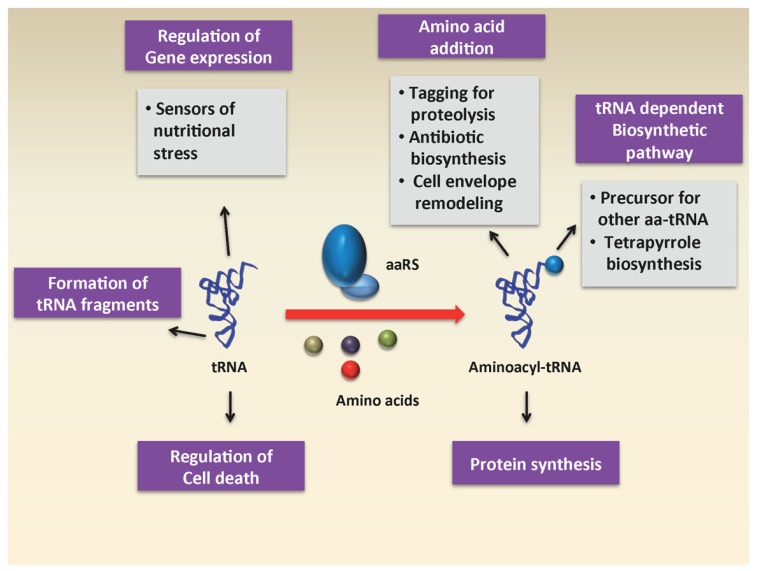
**Various roles of charged and uncharged tRNA in the cell**.

In recent years, the diverse roles of aa-tRNAs have received a great deal of attention. While much of the research has focused on the use of aa-tRNA by the ribosome for protein synthesis, a number of studies have uncovered roles for aa-tRNAs as substrates in other biochemical processes, such as cell wall formation, protein labeling for degradation, aminoacylation of phospholipids in the cell membrane, and antibiotic biosynthesis (**Figure 1**). In this section, we will briefly review some of these various processes that use aa-tRNAs as substrates.

### AMINOACYL-tRNAs IN CELL WALL BIOGENESIS

#### Aminoacyl-tRNA-dependent building of peptidoglycan bridges

Peptidoglycans (PG) are structural components of bacterial cell walls that can both serve as a barrier to environmental challenges and provide a scaffold for the attachment of various proteins including virulence factors (Vollmer et al., 2008). Peptidoglycan is a polymer of β (1-4)-linked *N*-acetylglucosamine (GlcNAc) and *N*-acetylmuramic acid (MurNAc), with all lactyl groups of MurNAc substituted with stem peptides, typically comprised of alternating D and L-amino acids with an overall common structure of L-Ala-γ-D-Glu-X-D-Ala-D-Ala. The composition of the peptide varies among different bacteria: Gram-negative bacteria and Gram-positive bacilli have meso-diaminopimelic acid (DAP) as the third amino acid (DAP-type peptidoglycan), whereas most other Gram-positive bacteria (including Gram-positive cocci) have L-lysine as the third amino acid (Royet and Dziarski, 2007). The stem peptides from adjacent strands are often crosslinked, either directly or through short peptides between the X position of the first pentapeptide side chain with the L-Ala at the fourth position of another. The amino acids required for bridge formation are typically derived from aminoacylated-tRNA donor molecules and are transferred onto the pentapeptide by tRNA-dependent aminoacyl-ligases which catalyze peptide-bond formation by using aminoacyl-tRNAs and peptidoglycan precursors as donor and acceptor, respectively.

The peptidoglycan in *Streptococcus pneumoniae* contains a “stem peptide” composed of up to five amino acids, Ala-γ-D-Glu-Lys-D-Ala-D-Ala, with an L-Ala-L-Ala or an L-Ser-L-Ala dipeptide branch that is attached to the third L-Lys of the pentapeptide side chain. MurM is responsible for the addition of either L-Ala or L-Ser as the first amino acid of the cross-link and then MurN invariably adds L-Ala as the second amino acid (Filipe et al., 2000). In both cases, appropriately aminoacylated-tRNA species serve as the amino acid donors for the reaction (Lloyd et al., 2008), although MurM also efficiently accepts mischarged tRNA substrates (Shepherd, 2011; Shepherd and Ibba, 2013b). In *Enterococcus faecalis*, BppA1 and BppA2 add L-Ala-L-Ala dipeptide to the pentapeptide chain (Bouhss et al., 2002), while FemXAB from *Staphylococcus aureus* sequentially adds one (FemX) or two (FemA and FemB) glycines (Schneider et al., 2004). Lif and Epr in *Staphylococcus simulans* and *Staphylococcus capitis*, FemX in *Weissella virides* and FemX and VanK in *Streptomyces coelicolor* all catalyze similar reactions using aa-tRNAs as substrates (reviewed in Shepherd and Ibba, 2013a). How aa-tRNAs are diverted from protein synthesis and used as substrates by these enzymes remains somewhat unclear in most instances. In *S. aureus*, the mechanism of escape from the protein synthesis machinery could be explained by the observation that three out of the five tRNA^Gly^ isoacceptors encoded in the *S. aureus* genome have sequence identity elements consistent with weak binding to EF–Tu (Giannouli et al., 2009). These specific tRNA sequence elements include replacement of the strong EF–Tu binding pairs G49–U65 and G51–C63 [23–25] with A49–U65 and A51–U63, respectively, in the T loop (Roy et al., 2007; Sanderson and Uhlenbeck, 2007). The three non-proteinogenic tRNA^Gly^ isoacceptors also show replacement of GG at positions 18 and 19 with either UU or CU. Hence the isoacceptors with weak binding to EF–Tu could escape protein synthesis and thus allow *S. aureus* to maintain an adequate supply of Gly-tRNA^Gly^ for two essential processes: translation and cell wall modification (Shepherd and Ibba, 2013a).

The specificity of peptidoglycan-modifying enzymes with respect to amino acid and tRNA substrates was demonstrated in the Fem X enzyme from *Weissella viridescens.* In *W. viridescens* the peptide bridge is made up of L-Ala-L-Ser or L-Ala-L-Ser-L-Ala. FemX initiates peptide bridge formation by transfer of the first L-Ala residue to the amino group of L-Lys found at the third position of the pentapeptide side chain. The enzymes involved in the subsequent transfer of the second position Ser and third position Ala residues have not yet been identified. FemX has a preference for L-Ala addition to UDP-MurNAc pentapeptide because it reacts much more unfavorably with both L-Ser and the acceptor arm of tRNA^Gly^. *In vitro* assays show that FemX turns over Ser-tRNA^Ser^ and Gly-tRNA^Gly^ 17- and 38-fold less efficiently than Ala-tRNA^Ala^, respectively. In the latter case, the penultimate base pair of tRNA^Ala^, G2-C71, was identified as an essential identity element for FemX. This is typically replaced by C2-G71 in tRNA^Gly^ species (Fonvielle et al., 2009). L-Ala is preferred 110-fold over D-Ala, suggesting relatively weak specificity toward different stereoisomers. The exclusion of serine is due to steric hindrance at the FemX_Wv_ active site rather than poor recognition of the nucleotide sequence of tRNA^Ser^. Hence, Fem enzymes discriminate non-cognate aa-tRNAs on the basis of both the aminoacyl moiety and the sequence of the tRNA.

#### Aminoacyl-tRNA-dependent aminoacylation of membrane lipids

Bacteria are frequently exposed to cationic antimicrobial peptides (CAMPs), for example eukaryotic host defense peptides or prokaryotic bacteriocins, whose cationic properties impart strong affinities to the negatively charged bacterial lipids phosphatidylglycerol (PG) and cardiolipin (CL). Many bacteria, among them several important human pathogens, achieve CAMP resistance using MprF proteins, a unique group of enzymes that aminoacylate anionic phospholipids with L-lysine or L-alanine, thereby introducing positive charges into the membrane surface and reducing the affinity for CAMPs (Ernst and Peschel, 2011). MprF was first identified when its inactivation rendered a *S. aureus* transposon mutant susceptible to a wide range of cationic antimicrobial peptides (CAMPs) leading to the name “multiple peptide resistance factor” (MprF; Peschel et al., 2001). MprFs can use lysyl or alanyl groups derived from aminoacyl tRNAs for modification of PG (Roy and Ibba, 2008). MprF proteins are integral membrane proteins made up of a C terminal, hydrophilic, cytoplasmic domain responsible for the transfer of amino acid onto PG, and an N terminal transmembrane hydrophobic domain that flips newly synthesized LysPG to the membrane outer leaflet (Ernst et al., 2009). MprF homologs can be found in most bacterial phylas and are abundant in firmicutes, actinobacteria, and proteobacteria with the exception of enterobacteria. Some archaea also harbor genes for MprF, probably resulting from lateral gene transfer events (Roy and Ibba, 2009). MprF homologs exhibit differential specificity for the aa-tRNA substrate they use to modify PG, resulting in a broader classification of these enzymes as aminoacyl-phosphatidylglycerol synthases (aaPGS; Klein et al., 2009; Dare and Ibba, 2012). For example, the MprFs in *S. aureus* and *P. aeruginosa* only synthesize Lys-PG or Ala-PG, respectively (Staubitz et al., 2004; Klein et al., 2009). In contrast, *Enterococcus faecium* MprF2 exhibits rather relaxed specificity for the donor substrate and produces both, Ala-PG and Lys-PG along with small amounts of Arg-PG (Roy et al., 2009). *Listeria monocytogenes* MprF is less strict in its specificity for the acceptor substrate and generates both, Lys-PG and Lys-CL (Thedieck et al., 2006; Dare et al., 2014). Based on the ability of MprF1 to efficiently recognize tRNA^Ala^, tRNA^Pro^, and a minihelix^Ala^ and recognition of the tRNA^Lys^ species from both *Borrelia burgdoferi* and humans, which share less than 50% sequence identity, it was proposed that the specificity of MprF arises from direct recognition of the aminoacyl moiety of aa-tRNA (Roy and Ibba, 2008). The mechanism utilized by MprF and other similar enzymes raises the question of how aa-tRNA donor substrates are directed into membrane lipid modification and away from protein synthesis. Determination of the *K*_D_s of Lys-tRNA for EF-Tu and for MprF suggested that the two proteins have similar affinities for tRNA under physiological conditions (Roy and Ibba, 2008). Comparison of the sites in tRNA recognized by MprF and EF–Tu would give a better understanding of how aa-tRNAs are partitioned between translation and membrane lipid modification pathways.

### ROLE OF AA-TRNA IN ANTIBIOTIC BIOGENESIS

In addition to having essential roles in protein synthesis and non-ribosomal peptide bond formation, aminoacyl-tRNAs are also used in pathways where the donated amino acid moiety undergoes transformation into a significantly different compound. These pathways involve different amino acid-tRNA pairs and a variety of acceptor molecules (Banerjee et al., 2010). Examples of aa-tRNA-dependent addition of amino acids in antibiotic biogenesis, which have been reviewed in detail previously, include valanimycin, pacidamycin, and cyclodipeptide synthesis (Shepherd and Ibba, 2013a).

Valanimycin is a potent antitumor and antibacterial azoxy compound first isolated from *Streptomyces viridifaciens* by Yamato et al. (1986). A gene cluster has been identified that contains 14 genes involved in the biosynthesis of valanimycin (Garg et al., 2002). The functions of the products of eight of these genes have now been established. Valanimycin is derived from L-Val and L-Ser via an isobutylhydroxylamine intermediate. VlmD, VlmH, and VlmR catalyze the conversion of valine into isobutylhydroxylamine, while VlmL catalyzes the formation of L-seryl-tRNA from L-serine. VlmA, which is a homolog of the housekeeping SerRS, catalyzes the transfer of L-serine from L-seryl-tRNA to isobutylhydroxylamine, to produce *O*-(L-seryl)-isobutylhydroxylamine, while VlmJ and VlmK catalyze the phosphorylation and subsequent dehydration of the biosynthetic intermediate valanimycin hydrate to form valanimycin (Garg and Parry, 2010). The mechanism by which Ser-tRNA^Ser^ is directed away from translation into the valanimycin pathway, and the identity elements of tRNA^Ser^ that help in recognition by VlmA and VlmL, are still unknown.

Other examples of antibiotics derived from aa-tRNAs are the cyclodipeptides (CDP), a large group of secondary metabolites with a notable range of clinical activities (Rodriguez and Carrasco, 1992; Prasad, 1995; Magyar et al., 1996; Waring and Beaver, 1996; Kanoh et al., 1999; Strom et al., 2002; Cain et al., 2003; Kanzaki et al., 2004; Jia et al., 2005; Kohn and Widger, 2005; Musetti et al., 2007; Minelli et al., 2012). It was originally proposed that formation of the CDPs was catalyzed by non-ribosomal peptide synthetases, which do not use aa-tRNAs as substrates. However, subsequent characterization of synthesis of the CDP albonoursin in *Streptomyces noursei* identified the tRNA-dependent CDP synthase AlbC (Lautru et al., 2002). AlbC synthesizes the albonoursin precursor cyclo (L-Phe-L-Leu) from aminoacylated tRNAs in an ATP-independent reaction (Lautru et al., 2002; Gondry et al., 2009). CDP synthase products identified to date include cyclo(L-Leu-L-Leu) (cLL), cyclo(L-Phe-L-Leu) (cFL), cyclo(L-Tyr-L-Tyr) (cYY), and cyclo(L-Trp-L-Xaa) (cWX), all of which are intermediates in antibiotic synthesis (Belin et al., 2012). CDP synthases use their two aa-tRNA substrates in a sequential ping-pong mechanism, with a similar first catalytic step: the binding of the first aa-tRNA and subsequent transfer of its aminoacyl moiety to the conserved serine residue of the enzyme pocket (e.g., Ser37 in the AlbC enzyme; Sauguet et al., 2011). The mechanism of addition of the second amino acid remains unclear, as do the specificity determinants for CDP synthases. Recently, similarities between the predicted secondary structure for PacB, a protein involved in the biosynthesis of the antibiotic pacidamycin, and structures of two Fem transferases led to the characterization of PacB as an alanyl-tRNA-dependent transferase (Zhang et al., 2011). Pacidamycins are a family of uridyl tetra/pentapeptide antibiotics produced by *Streptomyces coeruleorubidus* with antipseudomonal activities through inhibition of the translocase MraY during bacterial cell wall assembly. Analogous to the activity of CDP synthases, PacB hijacks aa-tRNAs and transfers L-Ala from aminoacyl-tRNA donors to the N terminal m-Tyr_2_ residue of the growing PacH-anchored antibiotic scaffold (Zhang et al., 2010).

### tRNA-DEPENDENT ADDITION OF AMINO ACIDS TO THE AMINO-TERMINUS OF PROTEINS

Protein degradation plays an important role in maintaining cellular physiology and in regulation of various cellular processes such as cell growth, differentiation and apoptosis by removing damaged polypeptides and regulatory proteins in a timely manner. As compared to cellular compartments like lysosomes and vacuoles where proteases are involved in non-specific degradation of proteins, protein degradation in the cytosol of prokaryotes and eukaryotes is often strictly targeted to protect cellular proteins from unwanted degradation. One means to achieve specificity involves the aa-tRNA transferases, which recognize a secondary destabilizing residue (pro-N degrons) at the N-terminus of a target peptide and utilize an aminoacyl-tRNA to transfer a primary destabilizing amino acid (N-degron) to the N-terminal residue, making the protein a target for the cellular degradation machinery (N-recognins; Mogk et al., 2007). This specificity in protein degradation was discovered by Bachmair et al. (1986) when they found that different genetic constructs of β-galactosidase proteins from *E. coli* exhibited very different half-lives when produced in *Saccharomyces cerevisiae*, ranging from more than 20 h to less than 3 min, depending on the identity of their N-terminal amino acid [the N-end rule (Bachmair et al., 1986)]. The N-end rule relates the identity of the N-terminal residue of a protein to its *in vivo* half-life (Mogk et al., 2007) and has been shown to function in bacteria (Tobias et al., 1991), fungi (Bachmair et al., 1986), plants (Potuschak et al., 1998) and mammals (Gonda et al., 1989). In eukaryotes an N-terminal Arg residue is the preferred N-degron and acts as a target for ubiquitin conjugation and subsequent degradation by the eukaryotic proteasome (Tasaki et al., 2012). The degron is generated by the *ATE1* gene product arginyl (*R*)-transferase, which transfers Arg from Arg-tRNA to the N-terminal α-amino group of oxidized cysteine, Asp, or Glu which constitute secondary destabilizing residues (Pro-N-degrons; Rai and Kashina, 2005; Graciet et al., 2006). In prokaryotes, Leu and Phe act as the primary destabilizing N-terminal residues (N-degrons) and can be generated by two classes of aa-transferases, leucyl/phenylalanyl(L/F)-transferase encoded by the *Aat* gene and leucyl- transferase encoded by *Bpt*. The L/F- transferase attaches a primary destabilizing residue of either Leu or Phe to the secondary destabilizing residues Lys, Met, and Arg (Shrader et al., 1993), whereas *Bpt*-encoded L-transferase attaches Leu to the secondary destabilizing residues Asp and Glu (Graciet et al., 2006). The Leu/Phe N-degron acts as a target for ClpS, which transfers the protein to ClpAP for subsequent degradation (Mogk et al., 2007). The question that next arises is how the aa-tRNA transferases achieve specificity in binding aa-tRNAs? The crystal structure of leucyl/phenylalanyl-tRNA-protein transferase and its complex with an aminoacyl-tRNA analog solved by Suto et al. (2006) revealed that the side chain of Leu or Phe is accommodated in a highly hydrophobic pocket, with a shape and size suitable for hydrophobic amino-acid residues lacking a branched β-carbon, such as leucine and phenylalanine. The adenosine group of the 3′ end of tRNA is recognized largely through π–π stacking with conserved Trp residues. However, L/F transferases achieve specificity for aa-tRNAs through specific interaction with the aminoacyl moiety and not the tRNA, and only the presentation of the specific aminoacyl moiety by a single-stranded RNA region is required for recognition (Abramochkin and Shrader, 1996). The activity of L/F-transferases is reduced in the presence of an excess of EF–Tu, suggesting that L/F-transferase and EF–Tu compete for binding to aa-tRNA.

## tRNA-DERIVED FRAGMENTS

**FIGURE 2:**
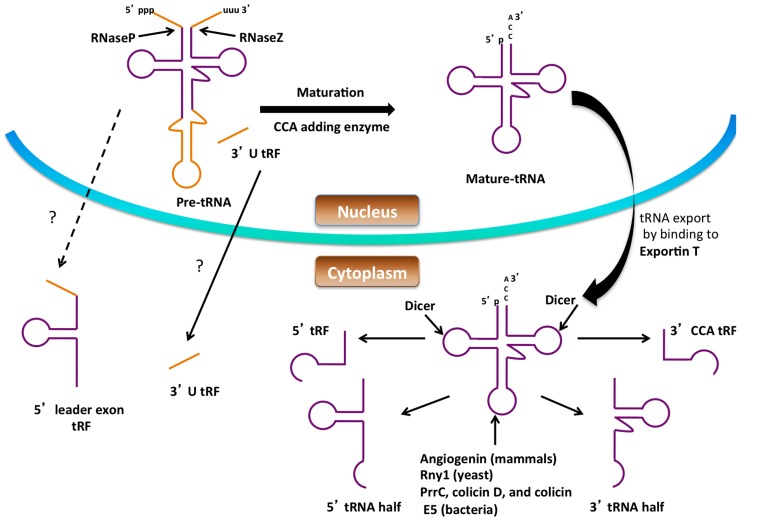
**Formation of small RNAs from tRNA**. Precursor tRNAs are processed by RNase P, RNase Z, the splicing endonuclease and CCA-adding enzyme to form mature tRNA in the nucleus. Processing of both the pre-tRNA and mature tRNA can give rise to small RNA. The figure shows possible routes for small RNA (tRNA halves, 5′ tRF, 3′ CCA tRF, 3′ U tRF and 5′ leader exon tRF) production from tRNA. The dashed lines and question marks indicate mechanisms of formation or transport of these tRFs that are not clear.

Small non-coding RNA (sncRNA) molecules are major contributors to regulatory networks that control gene expression, and significant attention has been directed toward their identification and studying their biological functions. sncRNA was first discovered in 1993 in *Caenorhabditis elegans*, and since then a large number of sncRNAs have been identified. sncRNAs are 16–35 nucleotides (nts) long and are classified into different groups such as microRNA (miRNA), small-interfering RNA (siRNA), piwi-interacting RNA, and small nucleolar RNA (snoRNA). Among them, miRNA and siRNA are the most extensively studied, and both suppress gene expression by binding to target mRNAs. The recent development of high-throughput sequencing technology has improved the identification of other types of small, RNAs-like, tRNA-derived RNA fragments (tRFs) which have been identified by several research groups (Lee et al., 2009). There is increasing evidence that these are not by-products from random degradation, but rather functional molecules that can regulate translation and gene expression. The production of tRNA fragments and their emerging roles in the cell are discussed below (**Figure 2**).

### PRODUCTION OF tRNA FRAGMENTS

#### tRNA halves

tRNA halves are composed of 30–35 nucleotides derived from either the 5′ or 3′ part of full-length, mature tRNA. These tRNA halves are produced by cleavage in the anticodon loop under nutritional, biological, physicochemical, or oxidative stress (Thompson et al., 2008; Dhahbi et al., 2013; Nowacka et al., 2013). In mammalian cells, tRNA halves are generated during stress conditions by the action of the nuclease angiogenin, a member of the RNase A family (Fu et al., 2009) whereas in yeast Rny1p, a member of the RNase T2 is responsible for tRNA half production. Apart from their roles as nucleases, both angiogenin and Rny1 act as sensors of cellular damage and can promote cell death and inhibit tumor formation (Thompson and Parker, 2009; Gebetsberger and Polacek, 2013). Under normal conditions, yeast Rny1 is usually localized in the vacuole (Thompson and Parker, 2009), while angiogenin is secreted to the plasma, sequestered in the nucleolus or bound to its inhibitor RNH1 (Yamasaki et al., 2009; Saikia et al., 2012), and released into the cytoplasm under certain stress conditions. tRNA halves have also been identified in bacteria, archaea, and plants. In bacteria, tRNA anticodon nucleases such as PrrC, colicin D, and colicin E5 have been shown to cleave specific subsets of tRNAs (reviewed in Kaufmann, 2000; Masaki and Ogawa, 2002).

#### tRNA-derived fragments

tRNA-derived fragments (tRFs) are shorter than tRNA halves ranging between 13 and 20 nt in size. They have been identified in all domains of life. There are four types of tRFs known and they are classified based on the part of the mature tRNA or pre-tRNA from which they are derived. tRFs were classified as 5′ tRFs, 3′ CCA tRFs, 3′ U tRFs, or 5′ leader-exon tRFs. 5′ tRFs are derived from the 5′ end of the tRNA generated at any point of tRNA processing, provided the 5′ leader sequence is removed by RNaseP, and are formed by a cleavage in the D loop. In the case of 5′ tRFs their biogenesis is carried out by Dicer in mammalian cells (Cole et al., 2009). However, it is known that the Dicer-independent generation of 5′ tRFs takes place in *Schizosaccharomyces pombe* due to the differences in length of the 5′ tRFs generated in these two organisms (19 nt long in mammals and 23 nt long in yeast), suggesting that a protein other than Dicer is responsible for their production in yeast (Buhler et al., 2008). 3′ CCA tRFs are produced from the 3′ ends of mature tRNA by cleavage at the T loop and carry the trinucleotide CCA at the acceptor stem. Dicer has been implicated in the generation of the 3′ end fragment (Maute et al., 2013), although angiogenin and other RNase A members have also been proposed to function in Dicer-independent processing (Li et al., 2012; Gebetsberger and Polacek, 2013). 3′ U tRFs are cleaved from the 3′ end of tRNA precursors by RNase Z, and their biogenesis is normally Dicer independent. They commonly start directly after the 3′ end of mature tRNAs and end in a stretch of U residues produced by RNA polymerase III run-off (Lee et al., 2009; Haussecker et al., 2010). One 3′ U tRF is produced in an RNaseZ-independent manner by the action of Dicer on the predicted bulged hairpin structure of the pre-tRNA (Babiarz et al., 2008). The mechanism of formation of 5′ leader-exon tRFs is not known; however they have been identified in CLP1 mutant cells possibly arising due to aberrant splicing. CLP1 is an RNA kinase and is a component of the mRNA 3′ end cleavage and polyadenylation machinery in mammals (Hanada et al., 2013).

While it was previously thought that production of tRNA halves and tRFs were solely mechanisms to remove damaged tRNAs, increasing evidence suggests their formation to be regulated. Angiogenin and Rny1 involved in the production of tRNA halves are usually sequestered in compartments before they are released in the cytoplasm where they cleave tRNAs (Spriggs et al., 2010). However, the regulation of their release from these cellular compartments is not known. Also a number of tRNAs [including tRNA^Asp(GTC)^, tRNA^Val(AAC)^ and tRNA^Gly(GCC)^] can be methylated by Dnmt2, which has been shown to protect these tRNAs from cleavage during stress (Schaefer et al., 2010). This specificity in cleavage of tRNAs might be responsible for the different types of tRFs observed under various conditions.

### FUNCTIONS OF tRFs

Are tRFs merely the products of tRNA degradation or do they have *bona fide* biological functions? If so, how diverse are these functions given the various forms of tRFs identified? Several lines of evidence point toward regulated production, suggesting that they may be functional RNA species. First, the abundance of different types of tRF does not correlate with the number of parent tRNA gene copies (Kawaji et al., 2008; Cole et al., 2009; Hsieh et al., 2009; Sobala and Hutvagner, 2011) with the exception of those found in *Tetrahymena* (Couvillion et al., 2010). Second, the fragments of tRNA formed are produced by cleavage at specific points in the tRNA. Third, whilst tRFs corresponding to the 5′ and 3′ ends of tRNA have been reported, those corresponding to the middle (incorporating the anticodon loop) have not. Although, the exact roles of tRNA halves and tRFs are yet to be elucidated, accumulating evidence suggests that tRNA-derived small RNAs participate in two main types of biological processes as discussed in more detail below.

#### Translation regulation of gene expression under stress conditions

tRNAs are indispensible components of the translational machinery, hence tRNA cleavage under stress conditions can affect protein synthesis. However, the mode of translational regulation by tRNA cleavage is not simple. It has been shown previously that during stress conditions, formation of tRNA cleavage products does not change the pool of full-length tRNA significantly, rather these fragments represent only a small portion of the tRNA pool (Saikia et al., 2012). Ivanov et al. (2011) showed a more intricate role for tRNA halves in translational control. They observed that tRNA halves formed by angiogenin during stress were able to inhibit protein synthesis and trigger the phospho-eIF2α-independent assembly of stress granules (SGs). These granules are mainly composed of stalled pre-initiation complexes, suggesting that the translation initiation machinery can be targeted by 5′ tRNA halves. They demonstrated that selected tRNA halves inhibit protein synthesis by displacing eIF4G/eIF4A from capped and uncapped mRNA and eIF4E/G/A (eIF4F) from the m^7^G cap. Using pull down of 5′-tiRNA^Ala^– protein complexes the authors implicated YB-1, a translational repressor known to displace eIF4G from RNA and eIF4E/G/A from the m^7^G cap (Evdokimova et al., 2001; Nekrasov et al., 2003). Analysis of the 5′ tRNA halves in complex with YB-1 revealed that a terminal oligo-G motif containing four to five consecutive guanosines present in certain 5′ tRNA halves (Ala/Cys) was absolutely required for translational repression of a reporter mRNA, suggesting the inhibition is caused by specific tRNA and is not a consequence of global upregulation of tRFs (Ivanov et al., 2011). This result came as a surprise as regulation of translation during stress is carried out via phosphorylation of eIF2 (See Roles of tRNA in Gene Expression), which induces translational repression facilitated by active sequestration of untranslated mRNAs into SGs (Holcik and Sonenberg, 2005).

In addition to tRNA halves, tRFs have also been implicated in regulation of translation. In the archaeon *Haloferax volcanii* a 26 nt-long 5′tRF originating from tRNA^Val^ in a stress-dependent manner was shown to directly bind to the small ribosomal subunit and inhibit translation by interfering with peptidyl transferase activity (Gebetsberger et al., 2012). A similar mechanism of translation inhibition by a 5′ tRF was recently observed in human cells (Sobala and Hutvagner, 2013). A 26 nt 5′ tRF derived from tRNA^Val^was able to inhibit translation by affecting peptide bond formation. An interesting observation from this study was that the tRFs required a conserved “GG” dinucleotide for their activity in inhibiting translation. A similar motif dependence is observed as discussed above in translation inhibition by a 5′ tRNA half. 5′ tRNA halves containing the 5′ tRF sequence were shown to require a run of at least four guanosine residues at the 5′ end of the molecule, which is present only in tRNA^Ala^ and tRNA^Cys^, as compared to 5′ tRFs that require only two guanosine residues at the 3′ end of the molecule, residues conserved between tRNAs. Mutating the di-guanosine motif required by 5′ tRF in the 5′ tRNA half did not affect its inhibitory activity, and the precise mechanism of translation inhibition by these tRFs warrants further investigation (Sobala and Hutvagner, 2013).

#### tRNA-derived fragments as regulators of gene silencing

One of the first studies showing the involvement of tRNA-derived fragments in gene regulation and silencing was carried out by Yeung et al. who addressed the role of small RNAs in human immunodeficiency virus (HIV) infected cells. A highly abundant, 18 nt-long, tRF originating from the 3′ end of human cytoplasmic tRNA^L^^ys3^ was shown to target the the HIV-1 primer-binding site (PBS) similarly to siRNAs that target complementary RNA (Yeung et al., 2009). tRNA^lys^ is used by viral reverse transcriptases as primer for the initiation of reverse transcription and DNA synthesis (Marquet et al., 1995). The 3′ tRF was shown to be associated with Dicer and AGO2, and to cause RNA cleavage of the complementary PBS sequence thereby showing the role of a tRF in viral gene silencing. Other tRFs like 3′CCA, 5′ and 5′ U tRF have also been shown to be associated with agronautes and hence have a potential to function as an siRNA or miRNA. Haussecker et al. (2010) investigated the ability of 3′ CCA and 3′U tRFs to associate with Argonaute proteins and cause silencing of a reporter luciferase transgene. They found that both types of 3′ tRF associated with Argonaute proteins, but often more effectively with the non-silencing Ago3 and Ago4 than Ago1 or Ago2. They observed that 3′ CCA tRFs had a moderate effect on reporter transgene silencing, but 3′ U tRFs did not. However, upon co-transfection of a small RNA complementary to the 3′ U tRF, the tRF preferentially associated with Ago2 and caused 80% silencing of the reporter transgene. This correlated with redirection of the reconstituted fully duplexed double-stranded RNA into Ago 2, whereas Ago 3 and 4 were skewed toward less structured small RNAs, particularly single-stranded RNAs. This is in stark contrast with results normally obtained in the miRNA field where sequences complementary to miRNAs relieve repression, a phenomenon known as sense-induced transgene silencing (SITS). Modulation of tRF levels had minor effects on the abundance of microRNAs, but more pronounced changes in the silencing activities of both microRNAs and siRNAs. This study provides compelling evidence that tRFs play a role in the global control of small RNA silencing through associating with different Argonaute proteins (Haussecker et al., 2010).

A tRF that functions as an miRNA was recently described, a 22 nt 3′tRF generated in a Dicer-dependent manner from tRNA^Gly^ in mature B cells and associated with Argonaute proteins (Maute et al., 2013). The 3′tRF was shown to inhibit RPA1, an essential gene involved in DNA repair by possibly binding to the 3′ UTR region. Expression of this tRF was downregulated in a lymphoma cell line indicating that loss of 3′ tRF expression might help the cancer cells to tolerate the accumulation of mutations and genomic aberrations during tumor progression.

#### Other biological functions of tRFs

Apart from the two known biological functions of tRFs in regulation, other potential biological functions are beginning to be identified. Recently a study by Ruggero et al. (2014) showed their role in viral infectivity. Large scale sequencing of small RNA libraries was used to identify small non-coding RNAs expressed in normal CD4^+^ T cells compared to cells transformed with human T-cell leukemia virus type 1 (HTLV-1), the causative agent of adult T-cell leukemia/lymphoma (ATLL). Among the miRNAs and tRFs expressed, one of the most abundant tRFs found was derived from the 3′ end of tRNA^P^^ro^, and exhibited perfect sequence complementarity to the primer binding site of HTLV-1. *In vitro* reverse transcriptase assays verified that this tRF was capable of priming HTLV-1 reverse transcriptase thereby suggesting an important role in viral infection. One possible role suggested for the tRF fragment is to support the initiation of reverse transcription, but not progressivity, with failure to proceed to the strand transfer step (Ruggero et al., 2014). Further studies are now needed to compare the abilities of the tRF and of full-length tRNA^Pro^ to prime and support strand transfer. Variation of tRNA halves accumulation was also shown in the parasites *Toxoplasma gondii*, the agent of toxoplasmosis, and the rodent malaria parasite *Plasmodium berghei*. These organisms exhibited increased tRNA accumulation upon egress from host cells and in response to stage differentiation, amino acid starvation, and heat-shock. It was observed that avirulent isolates of *T. gondii* and attenuated *P. berghei* parasites displayed higher rates of tRNA cleavage compared to virulent strains. Also tRNA half production was significantly higher in the metabolically quiescent bradyzoite and sporozoite stages of *T. gondii*, compared to the fast-growing tachyzoite indicating a relationship between half-tRNA production and growth rate in this important group of organisms (Galizi et al., 2013). A role for tRF halves in Respiratory Syncytial Virus (RSV) infectivity was recently shown by Wang et al. who observed an induction of tRNA cleavage upon RSV infection with a specific subset of tRNAs being cleaved. The 31 nt 5′ tRF(Glu) formed exhibited *trans*-silencing capability against target genes; however the mechanism of gene silencing was found to be different than the gene-silencing mechanism of miRNA/siRNA, previously also shown for other tRFs. Interestingly the tRF was also shown to promote RSV replication (Wang et al., 2013)

tRNA fragments have also been implicated in progressive motor neuron loss. Hanada et al. recently demonstrated that tRNA fragments generated in CLP1 mutant cells sensitize cells to oxidative stress-induced activation of the p53 tumor suppressor pathway and in turn lead to progressive loss of spinal motor neurons leading to muscle denervation and paralysis thereby providing a possible link between tRNA cleavage and p53 dependent cell death. However, the exact mechanism by which these tRNA fragments affect the p53 pathway needs to be determined (Hanada et al., 2013).

## REGULATION OF CELL DEATH BY tRNA

**FIGURE 3:**
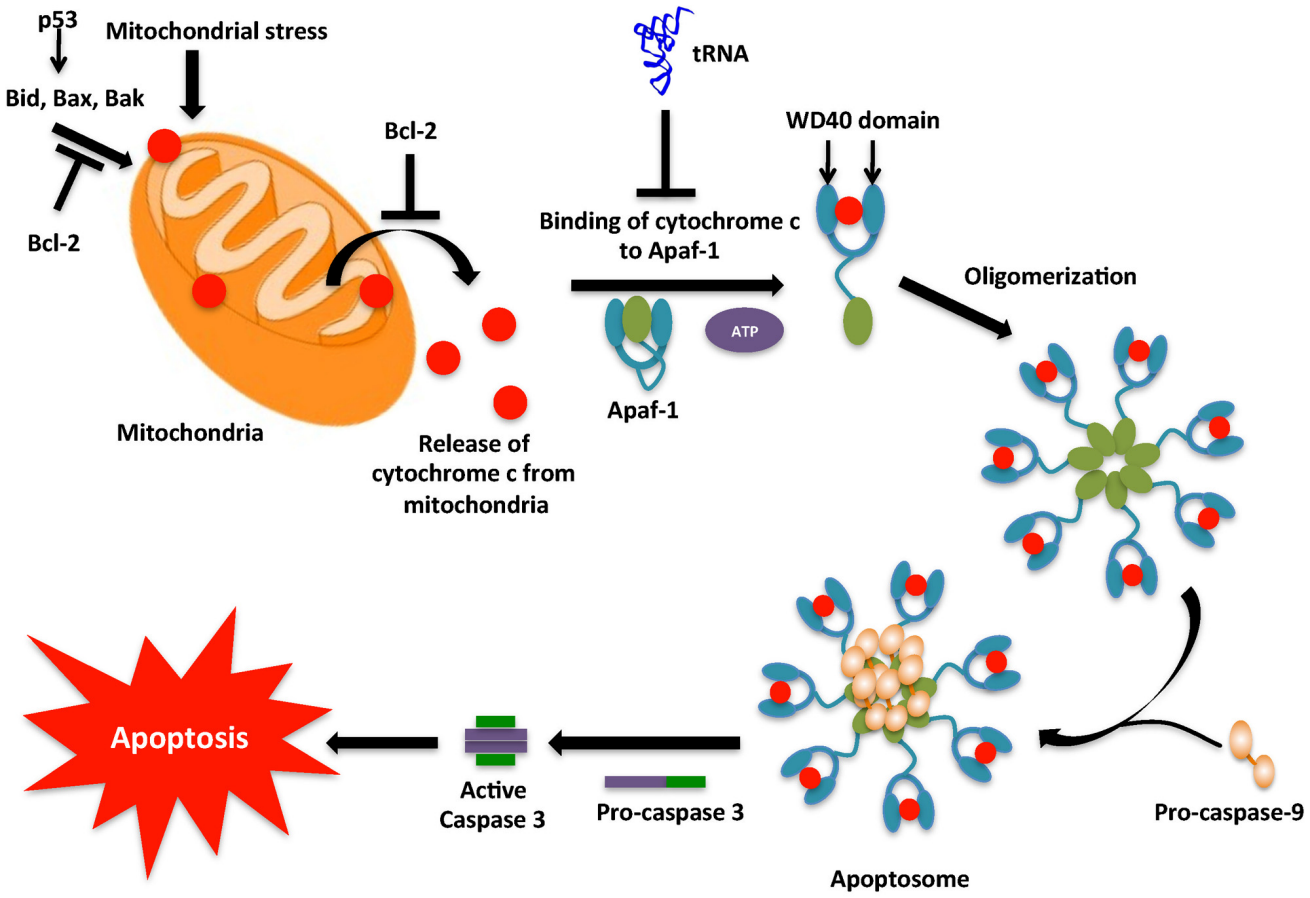
**Intrinsic pathway for apoptosis**. The intrinsic pathway, typically initiated by DNA damage activates p53. p53 then activates the pro-apoptotic proteins, which cause mitochondrial outer membrane permeabilization (MOMP) leading to release of cytochrome *c* into the cytoplasm. In the cytoplasm cytochrome *c* associates with Apaf-1 to form the apoptosome complex. However, tRNA may interact with cytochrome *c* and prevent its binding to Apaf-1. The apoptosome causes the conversion of inactive pro-caspase-9 into active caspase-9. Caspase-9 then activates caspase-3 that then leads to the caspase cascade, resulting in apoptosis.

Apoptosis is a cellular process by which damaged, harmful, and unwanted cells are eliminated. Apoptotic regulation is critical to cell homeostasis, immunity, multi-cellular development, and protection against infections and diseases like cancer (Thompson, 1995). Apoptotic cells have been shown to undergo various morphological and biochemical changes caused by a group of cysteine-dependent aspartate *s*pecific prote*ases*, or caspases. In healthy cells, caspases are inactive, however during apoptosis caspases are activated and signal the onset of apoptosis via cleavage of various intracellular proteins including apoptotic proteins, cellular structural and survival proteins, transcriptional factors, signaling molecules, and proteins involved in DNA and RNA metabolism (Li and Yuan, 2008; Hou and Yang, 2013). Cleavage of these intracellular proteins ultimately leads to phagocytic recognition and engulfment of the dying cell. While many factors have been discovered that regulate the apoptotic pathway, in this section the recently discovered role of tRNA as a regulator of cell death is discussed (**Figure 3**).

### CASPASE ACTIVATION BY EXTRINSIC AND INTRINSIC PATHWAYS

Apoptosis can be triggered via two major routes: an extrinsic, or extracellularly activated pathway and/or an intrinsic, or mitochondrial-mediated pathway. Both pathways activate caspases, a class of endoproteases that hydrolyze peptide bonds (Thornberry and Lazebnik, 1998). Although there are various types of caspases, those involved in apoptosis can be classified into two groups, the initiator (or apical) caspases and the effector (or executioner) caspases. Initiator caspases (e.g., Caspases-8 and 9) are capable of autocatalytic activation, whereas effector caspases (e.g., Caspases-3, 6 and 7) are activated by initiator caspase cleavage (Chang and Yang, 2000; Riedl and Shi, 2004). The extrinsic pathway begins outside the cell through activation of a group of pro-apoptotic cell surface receptors, such as Fas/CD95 and tumor necrosis factor receptor. Upon binding to their cognate ligand, these receptors recruit an adaptor protein Fas-associated death domain (FADD) that binds and dimerizes the initiator procaspase-8, to form an oligomeric death-inducing signaling complex (DISC), in which procaspase-8 becomes activated through an autoproteolytic cleavage event. The active caspase-8 then cleaves and activates the effector caspases 3 and 7 (Ashkenazi and Dixit, 1998; Krammer et al., 2007; Hou and Yang, 2013). The intrinsic pathway causes mitochondrial outer membrane permeabilization (MOMP), which leads to release of cytochrome *c*, a mitochondrial protein which transfers electrons from complex III to complex IV in the electron transport chain (Wang, 2001). The discovery of the role of cytochrome *c* in apoptosis by Liu et al. (1996) came as a surprise due to its essential role in the survival of the cell. In the cytosol, cytochrome *c* interacts with the apoptotic protease activating factor-1 (APAF-1) to form the apoptosome complex (Zou et al., 1997). The complex recruits procaspase-9, which converts to active caspase-9 by autocatalysis. Active caspase-9 activates effector caspases like caspase-7 and caspase-3 and causes apoptosis (**Figure 3**). Apoptosis is regulated by several pro-apoptotic proteins (Bax, Bak, and Bid), anti-apoptotic proteins (Bcl-2, Bcl-X_L_, and Mcl-1) and a range of cellular factors (HSP90, HSP70 and HSP27; Sreedhar and Csermely, 2004; Gorla and Sepuri, 2014) that is now known to include tRNA.

### INTERACTION BETWEEN tRNA AND CYTOCHROME c: POTENTIAL ROLE IN REGULATING APOPTOSIS

To answer the long-standing conundrum of why 1 mM dATP is required to induce caspase-9 activation in cell lysates, when the intracellular concentration of dATP is only 10 μM, Mei et al. investigated the role of RNA, which is essentially a polymer of nucleoside monophosphates, in cytochrome *c-*mediated caspase activation. They observed that treatment of mammalian S100 extracts with RNase strongly increased cytochrome *c-*induced caspase-9 activation, while the addition of RNA to the extracts impaired caspase-9 activation. These results implicated an inhibitory role of RNA in the activation of caspase-9. Systematic evaluation of the steps leading to caspase-9 activation identified cytochrome *c* as the target of the RNA inhibitor. Analysis of cytochrome *c*-associated species revealed that tRNA binds specifically to cytochrome *c*. Microinjection of tRNA into living cells inhibited the ability of cytochrome *c* to induce apoptosis, while degradation of tRNA by an RNase that preferentially degrades tRNA, onconase, enhanced apoptosis via the intrinsic pathway. Taken together, these findings showed that tRNA binds to cytochrome *c* and inhibits formation of the apoptosome (Mei et al., 2010). This suggested a direct role for tRNA in regulating apoptosis and revealed an intimate connection between translation and cell death. This finding also raised an interesting question as to how the interaction between tRNA and cytochrome *c* modulates apoptosis. This question was addressed recently by Gorla et al. who proposed that tRNA interacts with the heme moiety of cytochrome *c* and thereby protects the positively charged surface of cytochrome *c* from being exposed to the APAF-1 complex. This model was further confirmed by the observation that cytochrome *c* lost its ability to interact with tRNA after treatment with oxidizing agents or cysteine modifying agents. In such a state, hemin is unable to bind to tRNA and the exposed positively charged residues of cytochrome *c* then bind to APAF-1 (Gorla and Sepuri, 2014). Hence tRNA can regulate apoptosis by binding to cytochrome *c*. Further investigation of the nucleotide residues of tRNA involved in these interactions is required to answer questions about how tRNA binding to cytochrome *c* is regulated in the cell, whether specific tRNA isoacceptors are involved, and if this interaction is non-specific. Increased expression of tRNA has been detected in a wide variety of transformed cells (Marshall and White, 2008), such as ovarian and cervical cancer (Winter et al., 2000; Daly et al., 2005), carcinomas, and multiple myeloma cell lines (Zhou et al., 2009). Expression levels of tRNA molecules in breast cancer cells were 10-fold higher as compared to in normal cells and overexpression of tRNA_i_^Met ^induces proliferation and immortalization of fibroblasts and also significantly alters the global tRNA expression profile (Pavon-Eternod et al., 2013). It was also observed that certain individual tRNAs were overexpressed more as compared to others. tRNA^Arg(UCU)^, tRNA^Arg(CCU)^, tRNA^Thr (CGU)^, tRNA^Ser(CGA)^, and tRNA^Tyr(GTA)^ were among the most over-expressed tRNAs, while tRNA^His(GTG)^, tRNA^Phe (GAA)^, and tRNA^Met(CAT)^ were the least over-expressed tRNAs (Pavon-Eternod et al., 2009) indicating overexpression is not random and may be related to regulation of cytochrome *c*. Identification of the tRNA sites involved in binding to cytochrome *c* might help elucidate the connection between tRNA overexpression and cancer. tRNA cleavage has also been suggested as a mode of regulation of this interaction (Hou and Yang, 2013).

## CONCLUSION

While aa-tRNAs have been implicated in variety of roles in biosynthetic pathways, much less is known about the various functions of uncharged tRNAs in cells apart from their role in acting as sensors for cellular stress like nutritional deprivation. The recent discovery of the role of tRNA in regulating apoptosis has opened a whole new field which requires investigation into tRNA-protein interactions and has created a link between regulation of cell death and cellular metabolism. With the advent of high throughput sequencing techniques, studying the whole transcriptome of various organisms has become feasible. These techniques refuted the age-old assumption that rRNA, mRNA and tRNA constitute the main RNA species in the cell. It is now clear that almost all of the DNA in the cell is transcribed; however, only a small portion of these transcripts are translated into proteins or used as substrates for biological processes. The emergence of these sequencing techniques has resulted in discoveries of novel ncRNAs, and several studies have highlighted their role as important regulators of gene expression. Among the ncRNAs discovered, a number of cleavage products of tRNAs formed in response to stress have been also been discovered. These cleavage products were initially thought to be a result of random degradation; however, a number of studies have revealed their production to be a result of specific cleavage, and possibly regulated. Although a number of cleavage products have been observed, all the possible mechanisms of their production are not fully understood. Also in the case of 3′U tRF and 5′ leader exon tRF that are most likely produced inside the nucleus, their mechanism of export from the nucleus is not understood. Also regulation of tRNA fragment production, i.e., its initiation, efficiency, and termination are not fully understood. A recent study by Hanada et al. (2013) demonstrated that tRNA fragments sensitize cells to oxidative stress-induced activation of the p53 tumor suppressor pathway. This suggests that tRNA cleavage activates apoptosis via activation of p53 and hence protects against cancer, while full-length tRNA binds cytochrome *c* and prevents apoptosis thereby aiding cancer development. This hypothesis is strengthened by the overexpression of tRNAs observed in cancer cell lines. Further investigation into the link between tRNA cleavage and p53 activation is required to help understand how tRNAs help regulate the progression of cancer.

tRNA is post-transcriptionally modified at various nucleotides. While their role in tRNA structure stability and translation is well studied, these modifications might aid in the regulation of tRNA fragmentation. Further studies are needed to answer why some tRNAs are cleaved and others not – for example could modifications make certain positions in tRNA more sensitive to RNases or could they be responsible for blocking RNases? Also, modifications might also help regulate tRNA binding to cytochrome *c* during apoptosis. Regulation of this interaction and its role in metabolism and tumorigenesis will help our understanding of regulation of death in both normal and cancer cells. Cells have various mechanisms to sense the absence of a modification and remove non-functional tRNAs (Phizicky and Alfonzo, 2010). Variations in the modification status of tRNAs during stress have been implicated directly in decoding (Dedon and Begley, 2014), and such effects may be accentuated by indirect effects on the generation of regulatory tRFs. Clearly, much still remains to be discovered about the various regulatory roles of both charged and uncharged tRNA.

## Conflict of Interest Statement

The authors declare that the research was conducted in the absence of any commercial or financial relationships that could be construed as a potential conflict of interest.
